# The landscape of photoaging: From bench to bedside in a bibliometric analysis

**DOI:** 10.3389/fpubh.2022.972766

**Published:** 2022-10-20

**Authors:** Pei Hong Sun, Wei Tong Yan, Rui Feng Tian, Yan Sun, Yan Wu

**Affiliations:** ^1^Department of Dermatology, The First Hospital of China Medical University, Shenyang, China; ^2^Dalian Dermatosis Hospital, Dalian, China; ^3^NHC Key Laboratory of Immunodermatology, Ministry of Education Key Laboratory of Immunodermatology, National Joint Engineering Research Center for Diagnosis and Treatment of Immunologic Skin Diseases, The First Hospital of China Medical University, Shenyang, China; ^4^Department of Orthopedics, The General Hospital of Northern Theater Command, Shenyang, China

**Keywords:** bibliometric analysis, algorithm, ultraviolet, photoaging, knowledge structure, hotspots

## Abstract

**Background:**

Bibliometric software exists as a platform providing multiple algorithms to process the data to suffice diverse goals. Interpretation of the result must be based on insight into the meaning of the original data and the algorithm used. Medical Subject Headings (MeSH) terms represent the macro-level meaning of topics, keywords that commonly reflect the micro-level aspects.

**Objective:**

This study attempts to investigate the landscape of photoaging in the recent two decades by using bibliometric analysis.

**Methods:**

Published studies of photoaging were obtained from PubMed and Web of Science Core Collection (WoSCC) from 2000 to 2020. Basic bibliometric information was generated by WoSCC. Major MeSH terms were performed in cluster analysis and displayed as a hierarchical form to induce knowledge structure, detection algorithm on keywords was presented as a timeline form to obtain hotspots, and institutional clusters were labeled with keywords to achieve institutional characteristics.

**Results:**

A total of 2,727 and 2,705 studies were identified in PubMed and WoSCC, respectively. The number of photoaging-related studies at 3-year intervals grew steadily. The studies were performed in about 80 countries/regions. The highly frequent major MeSH terms were distributed in seven clusters, reflecting the etiology, pathophysiology, treatment, and prevention of photoaging. The hotspots changed as time went on, and the hotspots in recent 5 years were mitogen-activated protein kinase (MAPK), nuclear factor erythroid-derived 2-like 2 (Nrf2), and antioxidant activity. The highly productive institutions labeling in the top four clusters were Seoul National University, University of Michigan, China Medical University, and Harvard University, with corresponding keywords of UVB, retinoic acid, Nrf2, and rejuvenation.

**Conclusions:**

This study built a knowledge structure of pathophysiology, treatment and prevention of photoaging, and identified recent hotspots of MAPK, Nrf2, and antioxidant activity. We provide a landscape of photoaging, from the bench (pathophysiology) to bedside (treatment, prevention), and pave the way for future research.

## Introduction

Skin is a physical barrier between external and internal environments. It consists of cellular composition, structure proteins, and extrafibrillar matrix. Skin aging is a complex process caused by intrinsic and extrinsic factors. Intrinsic skin aging is a chronologically physiological change that results in senescence. Exogenous skin aging is mainly photoaging, with omnipresent solar ultraviolet (UV) radiation as the most important environmental factor. The UV-induced skin damages manifested as uneven pigmentation, telangiectasia, wrinkles, and laxity (1), were previously called skin premature. The explicit histologic characteristics and molecular mechanisms of UV-induced skin lead to the perception changes from skin premature to photoaging (2). Photoaging involves the superimposition of extrinsic radiation on a background of intrinsic skin aging. It may bring about artistic, psychological, or even life-threatening changes in human and is intervenable *via* sun protection, medicines, light/lasers, and so on. So far, photoaging is a continuous and increasing concern by researchers.

The data of published study vary from each other, such as authors, affiliations, keywords, publication years, and journals. These data are indexed in the database and located in a corresponding study. Basic bibliometrics function includes quantitative analysis of study productivities based on years, countries, journals, authors, institutions, and so on. Bibliometric analysis is a method to analyze published studies by using mathematical algorithms and statistical methods (3), and several important software platforms are available currently (4). By choosing specific algorithms on the platforms, the operators can extract different types of data and decode the results of algorithms with diverse goals. Knowledge structure and hotspots are “walls” and “bricks” of one topic, however, sometimes they are hard to define and obtain.

Medical Subject Headings (MeSH) is a comprehensively controlled vocabulary and special index of documents (studies) in PubMed (5). Every study can be indexed with several “major MeSH headings/subheadings” (simply called “MeSH terms” as follows) that represent the study topics. The study contents can also be indexed by keywords which are important words or brief phrases. Different from MeSH terms that usually designate macro-level branches of topics, keywords commonly reflect the micro-level issue. Take “photoaging” as an example, “Skin Aging/drug effects” is a MeSH term and “single oxygen” is a keyword. Under different algorithms, MeSH terms displayed in hierarchical form are expected to induce knowledge structure and keywords can be presented in timeline form to obtain hotspots.

It is necessary to perform a bibliometric analysis to obtain the research status of photoaging. However, the bibliometric analysis of photoaging has not been reported so far. In this study, the basic publication information, knowledge structure, hotspots, and institutional characteristics of photoaging were, respectively, identified by using this analysis. We provide a recent landscape of photoaging and a grasp of future research in this field.

## Materials and methods

### Data sources and search strategies

Published studies of photoaging were obtained from PubMed and Web of Science Core Collection (WoSCC) from January 2000 to December 2020. “Photoaging” and “TS = photoag^*^” was searched in PubMed and WoSCC, respectively. By choosing corresponding buttons, study types of articles and reviews were included. The articles included classical article, clinical study, clinical trial, comparative study, and randomized controlled trial. The reviews comprised review, systematic review, and meta-analysis. Other study types, for example, book and document, case report, comment, interview, letter, meeting (abstract), and so on, were excluded. The written language was confined to English.

### Data searching and collection

Data searching and collection were conducted by two independent reviewers (Peihong Sun and Ruifeng Tian). Disagreement was consulted by a third reviewer (Weitong Yan). In PubMed, the “citation information” including MeSH terms of each identified study was exported as a nbib formatted file. In WoSCC, the amount of studies sorted by years, countries/regions, journals, and authors were directly extracted from the search results, and the “full record and cited references” including keywords and institutions of each study were exported in text-format files.

### Bibliometric analysis

#### Basic publication information

Basic publication information of years, countries/regions, journals, and authors were directly extracted from the search result in WoSCC. The result of years was presented as a bar figure while those of countries, journals, and authors were presented as tables thereafter.

#### Knowledge structure

The nbib formatted file from PubMed was imported into the bibliographic item Co-occurrence Matrix Builder (BICOMB), a software used to construct the data matrix. Then, the MeSH terms and their frequency rankings were obtained. The MeSH term with ranking number *h* equal or less than appearance time *h* was defined as “highly frequent term.” Then, a binary matrix that had source studies as columns and highly frequent terms as rows was exported as an excel format file (Supplementary Table S1).

The binary matrix was imported into the graphical clustering toolkit (gCLUTO) version 1.0, a clustering software package that served as an easy-to-use platform (6). Clustering could be initiated by selecting the desired options with various algorithms along with analysis methods. Herein, the agglomerative algorithm of *I*_2_ was selected to implement the clustering process (6). The clustering results were presented as a mountain visualization along with a clustering table.

#### Hotspots

The text-format files from WoSCC were input to CiteSpace. Kleinberg's burst detection algorithm was selected to achieve increased burst keywords (7). These keywords frequently appeared within a certain period and were considered to be indicators of hotspots. The results were presented as lists with visualization bars.

#### Institutional characteristics

The text-format files from WoSCC were input to CiteSpace. The buttons of institutions and clustering analysis were chosen to achieve the clustering result of institutions (8). The noun phrases of keywords were used to label the institution clusters, and thus the distinct institutional characteristics were achieved.

## Result

**Figure 1:**
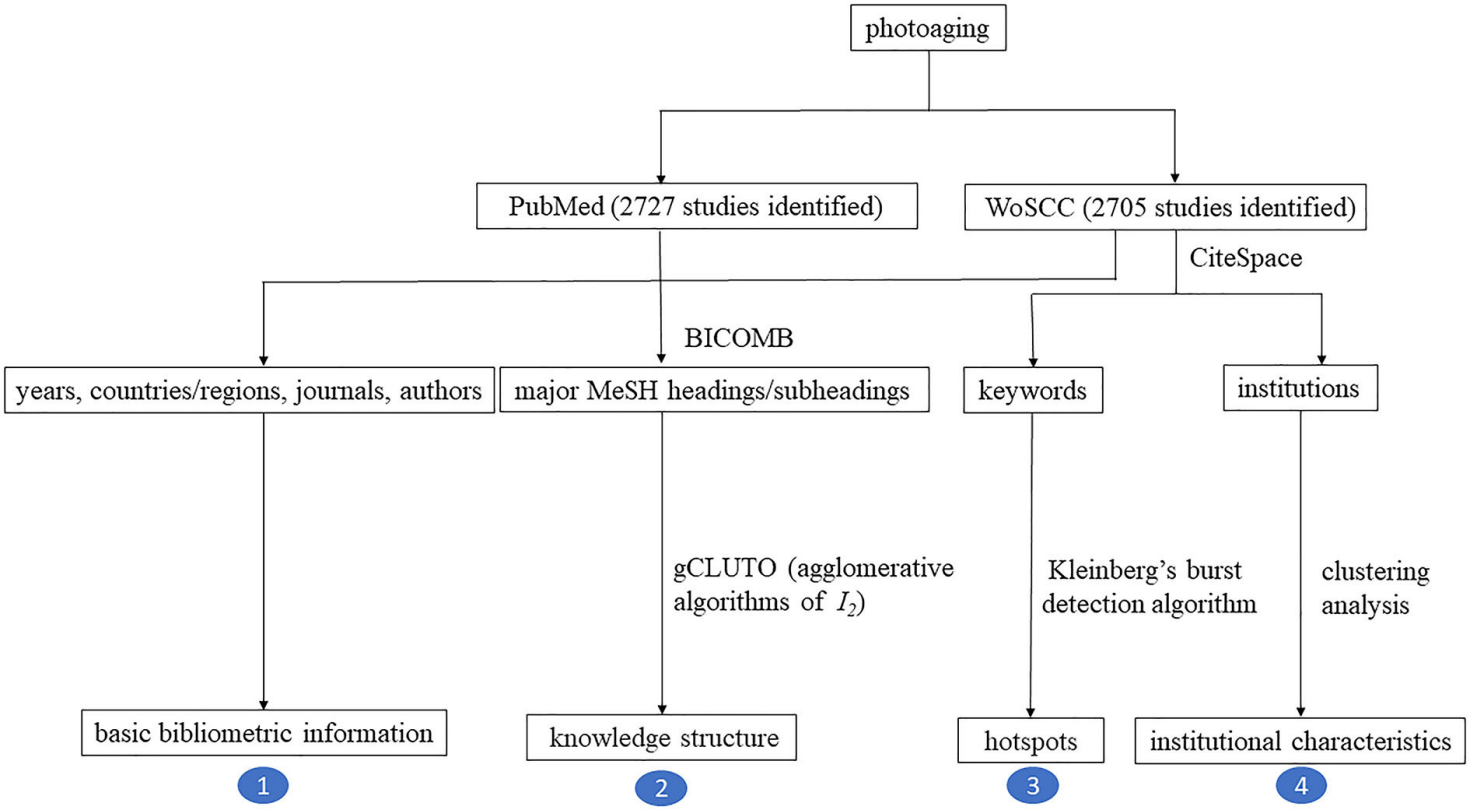
Flow diagram of bibliometric analysis strategy.

In PubMed, after the exclusion of 105 ineligible studies, a total of 2,727 studies (2,233 articles and 494 reviews) were identified. In WoSCC, 2,705 studies (2,383 articles and 322 reviews) were identified. The flow chart of bibliometric variables and corresponding algorithms are shown in Figure 1.

### Basic publication information

**Figure 2:**
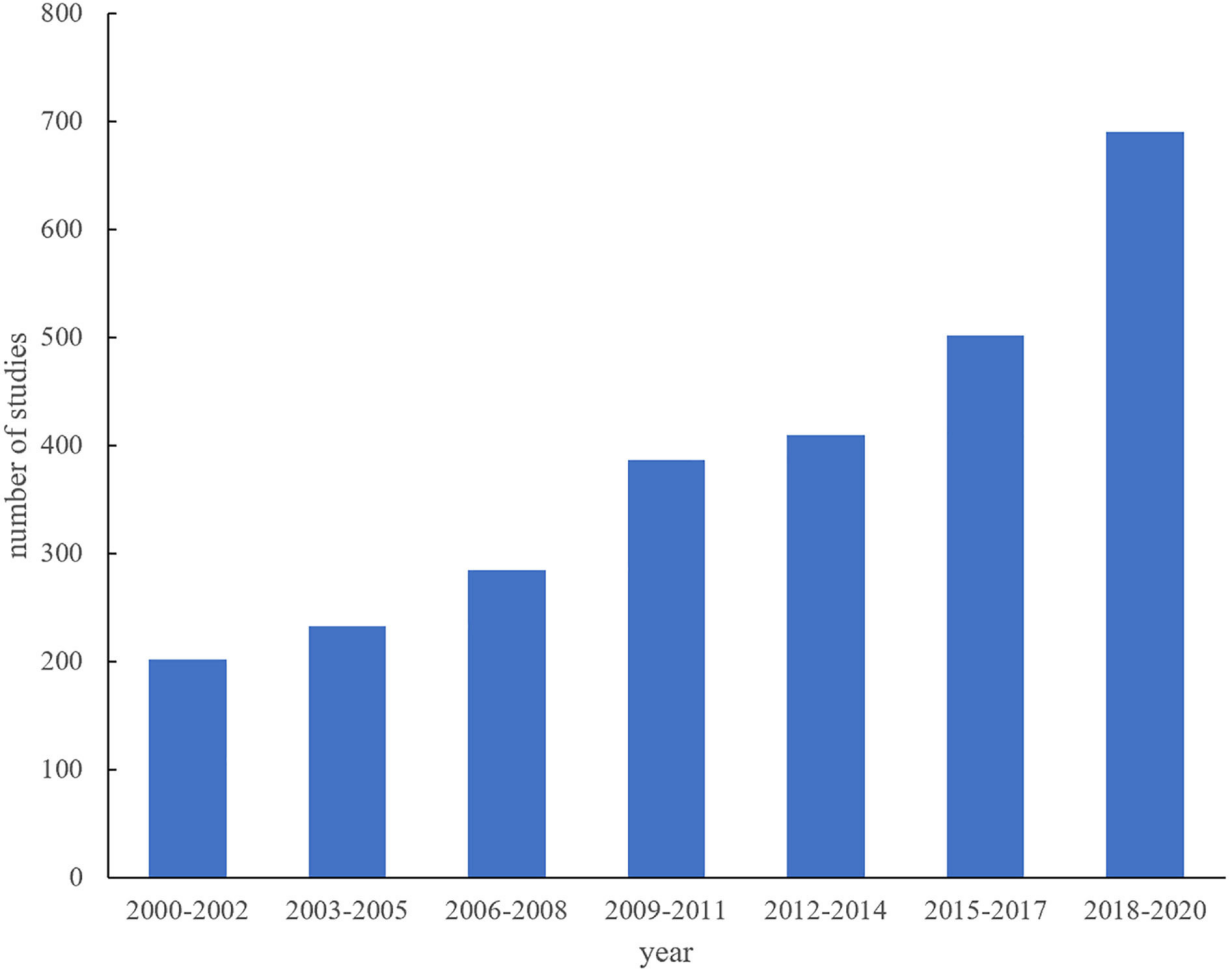
Numbers of published photoaging studies from 2000 to 2020 at 3-year intervals.

Bibliometric analysis of years, countries/regions, authors, and journals were originated from WoSCC. The number of photoaging-related studies at 3-year intervals grew steadily over the past two decades (Figure 2). The studies were performed in about 80 countries/regions. USA (*n* = 769) was the biggest contributor to photoaging, followed by South Korea (*n* = 519), China (*n* = 321), Japan (*n* = 225), France (*n* = 208), and so on. The top 10 active journals accounted for 26.98% (730/2,705) of the included studies. The prolific researchers were Chung JH, Hwang E, Fisher GJ, Yi TH, Voorhees JJ, and so on. The top 10 active countries/regions, journals, and authors in photoaging research were presented in Table 1.

**Table 1:** Top 10 active countries/regions, journals, and authors in photoaging research.

**Rank**	**Country/region**		**Journal**		**Author**	
	**Country/region**	**Study count**	**Journal title**	**Study count**	**Author**	**Study count**
1	USA	769	Dermatologic Surgery	102	Chung JH	69
2	South Korea	519	Journal of Cosmetic Dermatology	82	Hwang E	41
3	China	321	Journal of Drugs in Dermatology	81	Fisher GJ	40
4	Japan	225	Photochemistry and Photobiology	74	Yi TH	40
5	France	208	Experimental Dermatology	73	Voorhees JJ	38
6	Germany	151	Journal of Investigative Dermatology	72	Krutmann J	31
7	England	118	Journal of Dermatological Science	70	Griffiths CEM	28
8	Brazil	117	British Journal of Dermatology	69	Kang S	26
9	Italy	104	Journal of Photochemistry and Photobiology B Biology	58	Kim KH	24
10	Taiwan	66	Photodermatology Photoimmunology Photomedicine	49	Lee DH	24

### Knowledge structure

**Figure 3:**
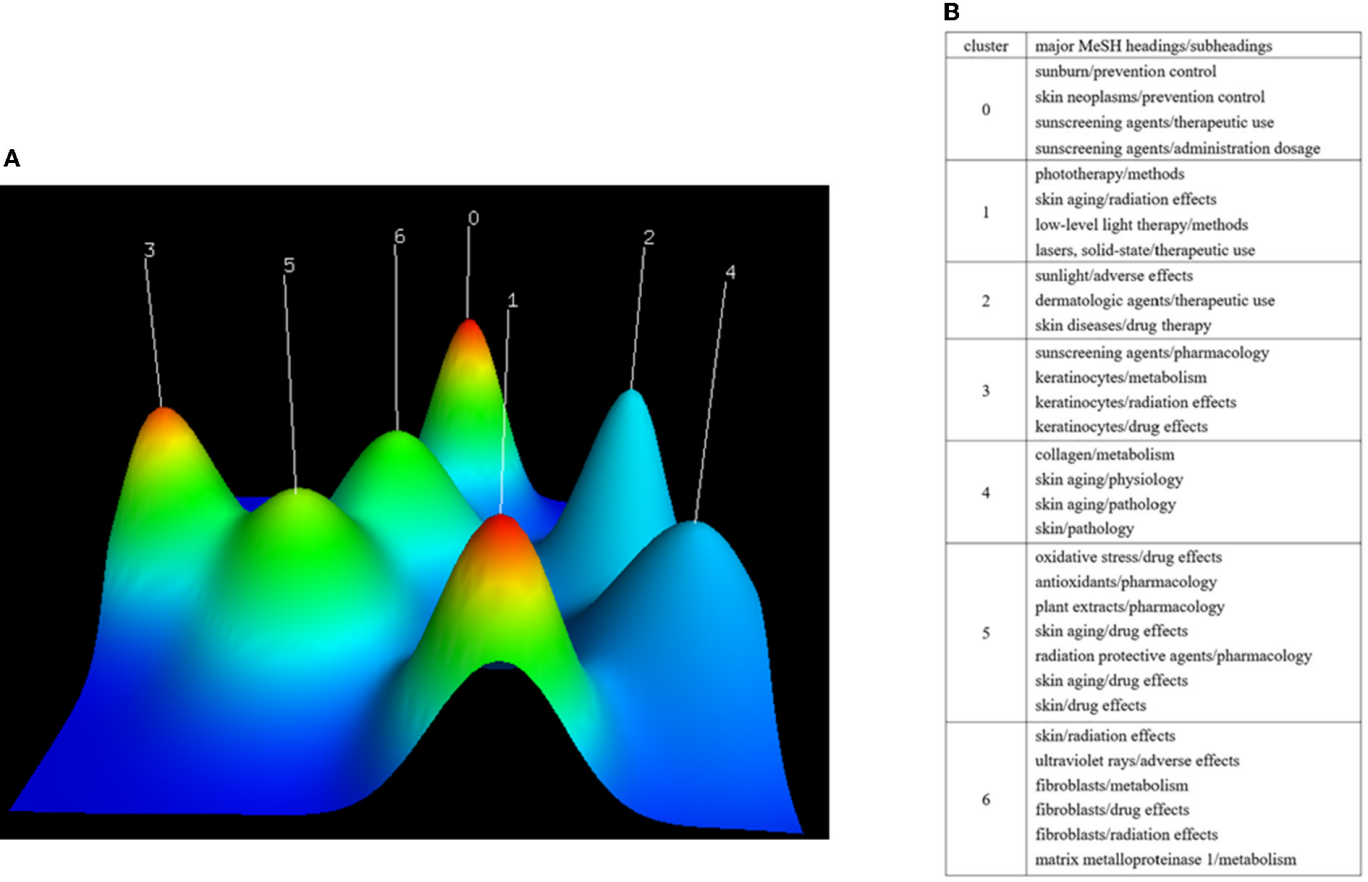
Clusters of highly frequent major MeSH headings/subheadings presented in mountain visualization along with a detailed clustering table. **(A)** In mountain visualization, each mountain represents a cluster, height of the peak represents internal similarity in the cluster, red color indicates a low deviation and blue color indicates a high standard deviation, and the volume of each mountain represents the number of highly frequent major MeSH headings/subheadings in the cluster. **(B)** The detail of highly frequent major MeSH headings/subheadings in each cluster.

A total of 2,734 MeSH terms were obtained from PubMed. The cumulative frequency of all MeSH terms was 9,283. There were 32 highly frequent MeSH terms and the cumulative percentage of them was 36.07% (3,349/9,283). The 32 MeSH terms were contained in 63.87% (1,742/2,727) studies and distributed in 7 clusters presented in mountain visualization. In the detailed clustering table, information on prevention of photoaging (cluster 0), treatment of photoaging (cluster 1), UV and skin diseases (cluster 2), keratinocytes and photoaging (cluster 3), pathophysiology of photoaging (cluster 4), antioxidant treatment of photoaging (cluster 5), and fibroblasts and photoaging (cluster 6) were separately categorized into the seven clusters (Figure 3).

### Hotspots

**Figure 4:**
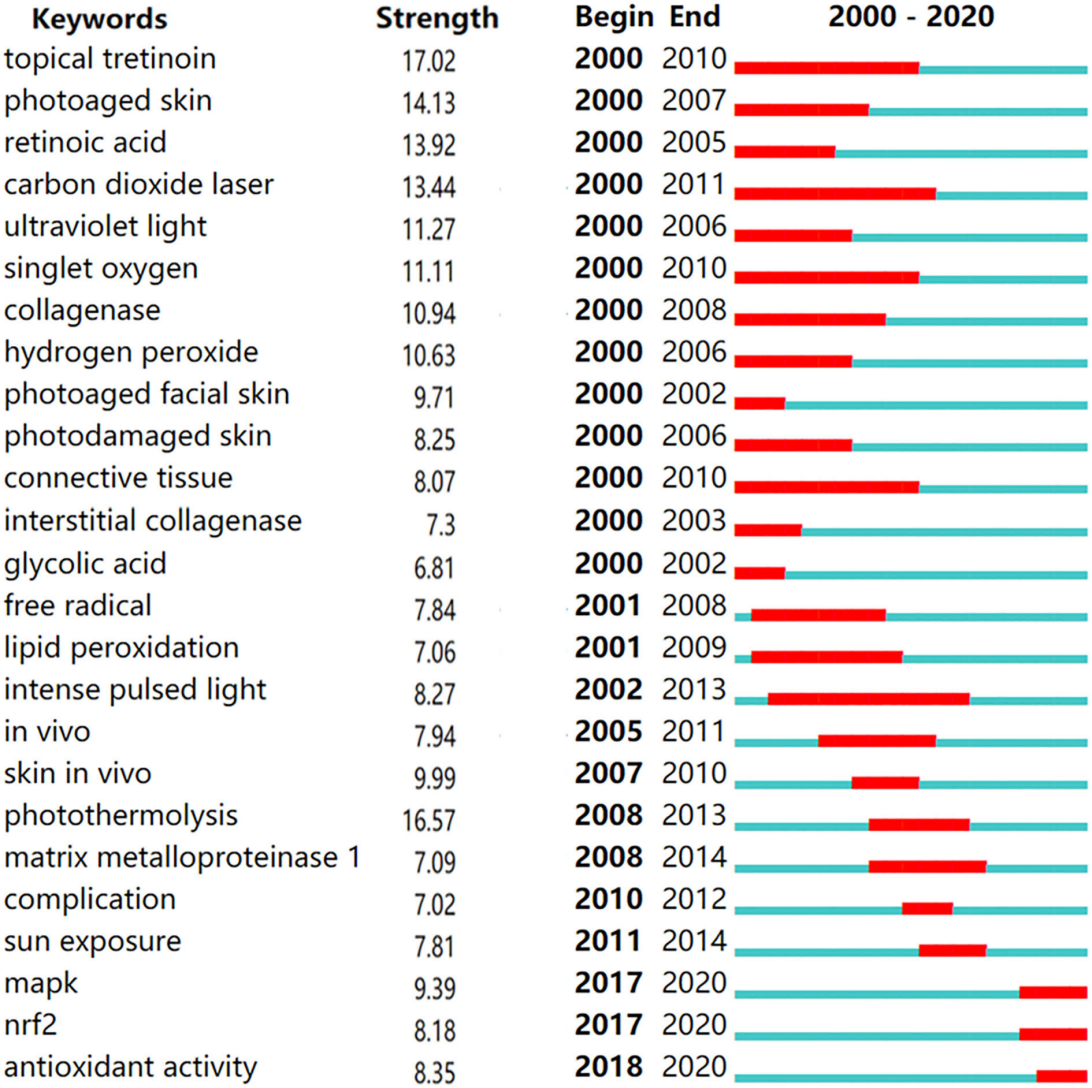
Hotspots of photoaging studies from 2000 to 2020. Red bar represents the duration that burst keywords last.

The hotspots in photoaging changed, as the burst durations of the top 25 keywords which had strong citations changed, from 2000 to 2020 (Figure 4). The burst keywords covered mechanism, treatment, and prevention of photoaging. The top five burst keywords were topical tretinoin, photoaged skin, retinoic acid, carbon dioxide laser, and UV light. However, they were no longer hot in the recent decade. The recent burst keywords, that is, recent hotspots, were mitogen-activated protein kinase (MAPK), nuclear factor erythroid-derived 2-like 2 (Nrf2), and antioxidant activity, presenting as high burst in the recent 5 years.

### Institutional characteristics

**Figure 5:**
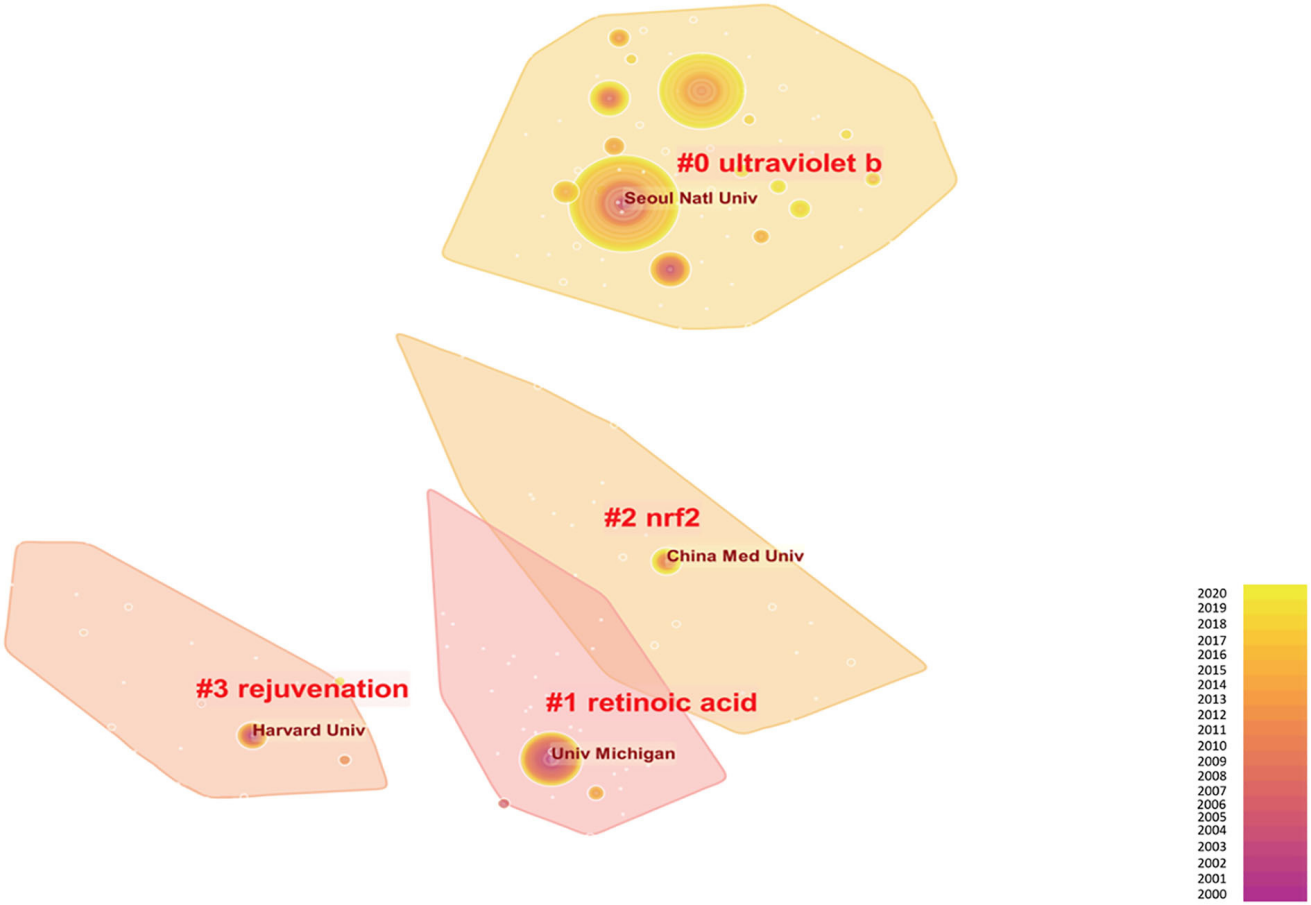
Institutional analysis labeled with different keywords of photoaging. Size represents the study productivity of the institution, that is, the bigger the size, the more productivity of the institution; colors from purple to yellow represent publication chronology from 2000 to 2020.

Institutional clusters were labeled with keywords and there were a total of 229 clusters in the network. The highly productive institutions labeling in the top four clusters were Seoul National University, University of Michigan, China Medical University, and Harvard University. Correspondingly, the keywords labeling them were ultraviolet B, retinoic acid, Nrf2, and rejuvenation. University of Michigan and Harvard University were active in their early years, and Seoul National University and China Medical University published more studies recently. Figure 5 showed the top four institutions and their characteristics.

## Discussion

From 2000 to 2020, publications of photoaging present a continuously rising tendency, indicating that photoaging always attracts the attentions of researchers, especially in recent 3 years. The USA contributed the most in this field as it ranked No.1 in terms of the number of publications, the next was South Korea, and the other two Asian countries (China and Japan) also contributed a lot. Among the top 10 journals, the top four were from the USA, the other four were from England, and another two were, respectively, from Ireland and New Zealand. The top 10 authors came from 5 institutions, with 2 institutions located in South Korea and the other 3 in the USA, England, and Germany, respectively. The authors from the same institutions may have the same interesting study orientations or have overlapped study contents.

**Figure 6:**
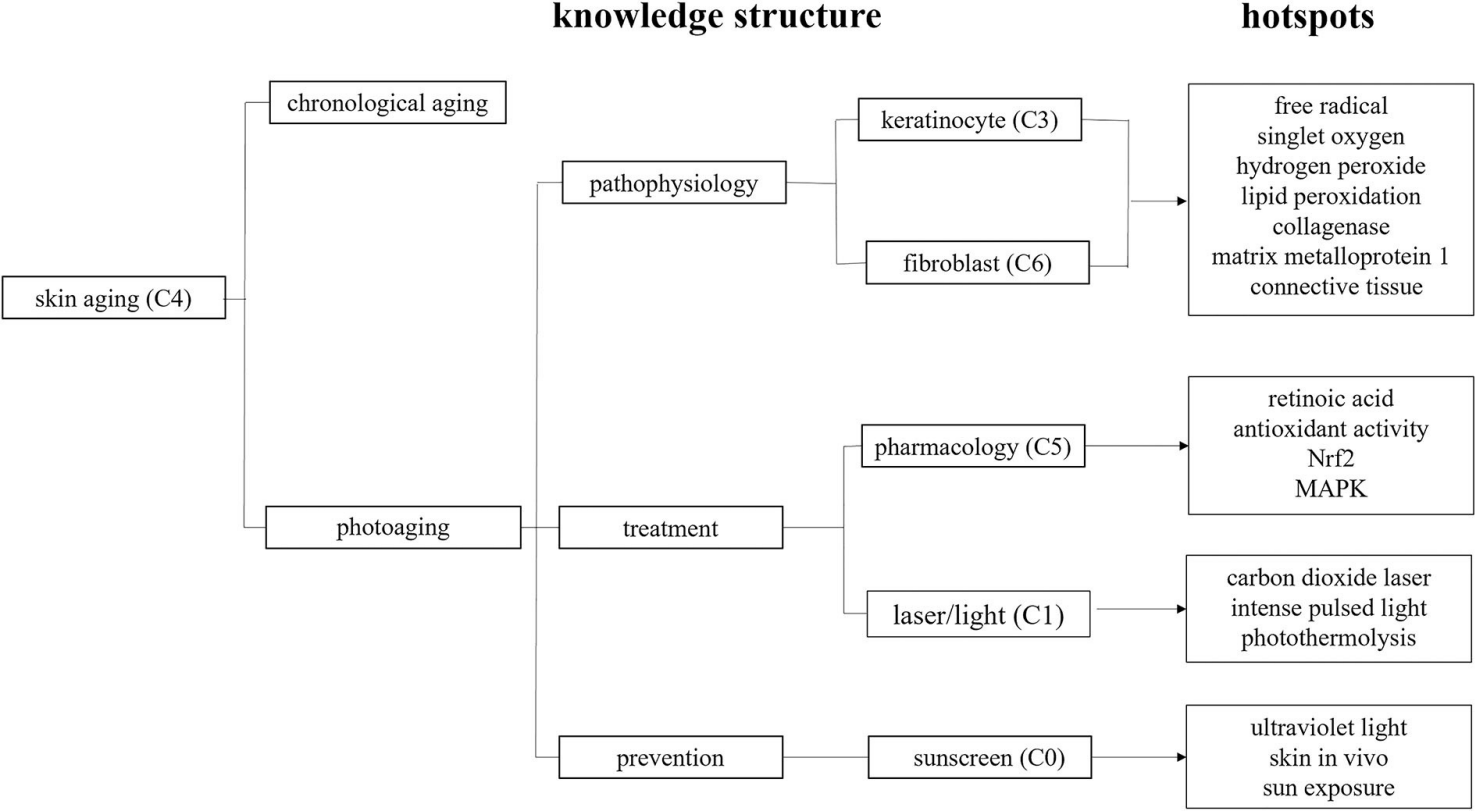
Panorama of photoaging studies deducing from knowledge structure and hotspots. C, cluster, for example, C1 representing cluster 1; MAPK, mitogen-activated protein kinase; Nrf2, nuclear factor erythroid-derived 2-like 2.

For a topic, such as photoaging, the study contents can be indexed by MeSH terms and keywords. Interpretation of the results originating from specific algorithms should be based on insight into the meaning of MeSH terms and keywords. On one hand, the knowledge structure coming from MeSH terms can be categorized into a common paradigm of disease (a topic), for example, etiology, pathophysiology, clinical appearance, treatment, prevention, and so on. On the other hand, the hotspots deriving from keywords can show details of the knowledge structure. In the present study, the knowledge structure and hotspots were summarized as the outlines of pathophysiology, treatment, and prevention of photoaging, thus giving a landscape of photoaging (Figure 6), from the bench (pathophysiology) to bedside (treatment, prevention). The bibliometric algorithms are routinely used in bibliometric analysis. Herein, we combined them with other available information to build a vivid landscape visualized in Figure 6, which is an innovative idea.

### Pathophysiology

The knowledge structure and hotspots portrayed a cellular and molecular map for us to understand the reactive oxygen species (ROS) that started the cascade signaling pathway. UV-induced overproductions of singlet oxygen, hydrogen peroxide, lipid peroxidation, and so on (9, 10) affect tissue homeostasis by influencing cell signaling and phenotype (2, 11). Matrix metalloprotein 1 (MMP1), a kind of collagenases, was once the research hotspot during 2008–2014 (12–16). It is now clear that UV-induced overexpression of collagenases degrades dermal connective tissue. The mechanisms that UV-induced skin connective tissue damages comprise cell surface receptors, protein kinase signal transduction pathways, transcription factors, enzymes, and so on (13, 17–19). Various cells and molecules are involved in these processes (16), and in our study, knowledge structure emphasizes the cellular elements of photoaging and hotspots underline the molecular components.

### Treatment

According to the knowledge structure, the treatment of photoaging consists of pharmacology and laser/light modalities. From the burst detection, we can see that retinoic acid was one of the hotspots in the early years. Tretinoin, the most commonly used retinoic acid in photoaging, has been approved by Food and Drug Administration for decades. However, cutaneous irritation limited the widespread use of retinoic acid. The hotspots changed as time went on. Antioxidant activity, Nrf2, as well as MAPK, became recent hotspots. As ROS plays an important role in initiating the cascade signal pathways in photoaging, antioxidant therapy is an important strategy to combat the situation. The skin's intrinsic ROS defense equipment consists of detoxifying enzymes and antioxidant molecules (20). Superoxide dismutase, catalase, and glutathione peroxidase are important enzymes that help ROS convert into harmless materials. An antioxidant molecule is a material that can reduce oxidative stress through electronic transfer way. When an intrinsic antioxidant system cannot overwhelm the photodamages caused by UV, exogenous antioxidant substances are needed to supplement (21). In our study, plant extracts appeared in cluster 5. The emerging active ingredients, include equol (22), neferine (23), ginsenoside Rg2 (24), mangiferin (25), genistein (26), sesamol (27), vicenin-2 (28), and so on. They counteract with UV-induced oxidative stress by promoting the activity of antioxidant enzymes and reducing the oxidative stress-induced oxidations of DNA, protein, and lipid. Moreover, two phytochemical constituents, pterostilbene and curcumin, exhibit increased expression of Nrf2 (29, 30). In response to UV-induced oxidative stress, Nrf2 induces the expression of cytoprotective proteins, including detoxifying enzymes and antioxidant proteins. A recent report showed that Nrf2 is involved in mottled pigmentation of photoaging skin (31), and it is now recognized as a potential target for drug development. The photoprotective effects of pharmacological activation of Nrf2 are verified in experiments (32). MAPK is the important downstream of the ROS signaling pathway. The role of MAPK in the mechanism of photoaging has been clearly stated many years ago (2); however, it becomes a hotspot in recent years. The possible reason for the revival is that the MAPK pathway is usually regarded as the criteria marker in current antiaging research (33–35). Laser/light-based rejuvenation, *via* selective photothermolysis, remains an important aesthetic tool for photoaging skin (36). Our study detected two hotspots, that is, carbon dioxide (CO_2_) laser and intense pulsed light (IPL), representing two classical ablative and non-ablative modalities. CO_2_ laser has been recognized as the “golden standard” for rejuvenation and popular for a long time. For IPL, people would like to choose it for the diverse indications with limited side effects, especially after the innovation of IPL technology from “V shape beam” to “square shape beam” in 2000 (37).

### Prevention

In addition to the generation and accumulation of ROS contributing to the photoaging, prolonged and repeated exposure to UV radiation also causes genetic changes (38) and immune system suppression (39–41). Such multiple insults inflict on the skin over time and ultimately lead to malignant/premalignant neoplasms (photo-carcinogenesis) (42). Therefore, sunscreen is not only related to youthful appearance but also to health problems. It should be reminded that the sun also includes visible light and infrared light which may play a vital role in photoaging and photodamage (43, 44). Photoprotection of this wave band is a new challenge in a sunscreen product, maybe using tinted sunscreen is an applicable choice (45). Evidence suggested that daily sunscreen use is important for the prevention of photoaging. Even more, protection from the sun at any time reduces the risk of actinic keratoses and squamous cell cancer (46).

### Institutional characteristics

Of the top four institution clusters, the University of Michigan contributed the most to the field of photoaging in the previous decade. The majority of important studies (having a high index factor or being cited a lot by others) were from this famous institution. The University of Michigan was labeled with hotspot retinoic acid, as the anti-aging mechanism and prevention effects of retinoic acid were especially elaborated by Fisher et al. in the institution (13, 47–49). Other substantial photoaging researches concerning procollagen biosynthesis, collagen degradation, and signal transduction (18, 50–54) were also from the University of Michigan. The early studies have shaped vital cognitions of photoaging and built a cornerstone for current research. An institution cluster was labeled with another hotspot Nrf2. China Medical University located in Taiwan contributed the most and was active in the recent decade in this aspect. Nrf2 is one of the factors to maintain the homeostasis of oxidative stress and the antioxidant system. Taking advantage of traditional Chinese medicine, including plant extracts, the institution revealed the antiaging mechanisms through the Nrf-2 pathway (55–58). A mass cluster was labeled with UVB. In this aspect, Jin et al. from Seoul National University College of Medicine had a large number of publications on UVB-induced premature aging and anti-aging mechanisms (59–62). The fourth institution cluster was labeled with rejuvenation, which has long been a focus on photoaging treatment by laser/light. Of note, Harvard university raised the fractional photothermolysis concept to minimize side effects, which greatly expanded the scope of laser application (63).

### Limitations

First, photoaging itself is not a MeSH in PubMed. However, the “major MeSH headings/subheadings” relating to photoaging convey the diversity and plentiful meanings, and we could build a relatively integrated knowledge structure of photoaging. Second, the studies included were from 2000 to 2020. Studies from some institutions, for example, the University of Michigan, provided compelling evidence and research on the mechanism of photoaging traced before 2000 (13, 47, 64). Third, even though there were similar amounts of studies in PubMed and WoSCC, they may have some differences to lead to deviated results. Finally, not all factors, for example, co-occurrence of cited articles, networks of authors/institutions/countries, and so on, were chosen in our bibliometric analysis.

## Conclusion

Using bibliometric analysis, this study built a knowledge structure of pathophysiology, treatment and prevention of photoaging, and identified recent hotspots of MAPK, Nrf2, and antioxidant activity. Furthermore, combining both of them, we got a landscape of photoaging, from the bench (pathophysiology) to bedside (treatment, prevention). Nowadays, bibliometric software is popular and convenient, which can be used to decode complicated information on a topic. Deriving the knowledge structure and hotspots may become a universality analysis strategy to help us quickly grasp the “walls” and “bricks” of one topic.

## Data availability statement

The original contributions presented in the study are included in the article/Supplementary material, further inquiries can be directed to the corresponding author.

## Author contributions

PS: data entry and management, draft the article and selection of manuscripts to discuss the results, and analysis and interpretation of data. WY: conception and design of the study, acquisition, analysis, and interpretation of data. RT: selection of manuscripts to discuss the results and revising them critically for important intellectual content. YS: final editing for corrections in the English quality and final approval of the version to be submitted. YW: acquisition, analysis, interpretation of data, revising it critically for important intellectual content, final editing, and final approval of the version to be submitted. All authors read and approved the final manuscript.

## Funding

The work was supported by the National Natural Science Foundation of China (Grant number 82103758).

## Conflict of interest

The authors declare that the research was conducted in the absence of any commercial or financial relationships that could be construed as a potential conflict of interest. The reviewer LT declared a shared affiliation with the authors to the handling editor at the time of review.

## Publisher's note

All claims expressed in this article are solely those of the authors and do not necessarily represent those of their affiliated organizations, or those of the publisher, the editors and the reviewers. Any product that may be evaluated in this article, or claim that may be made by its manufacturer, is not guaranteed or endorsed by the publisher.
