# Supplementary material for: The landscape of photoaging: From bench to bedside in a bibliometric analysis
Source: Front Public Health. 2022 Oct 20;10:972766. doi: 10.3389/fpubh.2022.972766 (PMC9633272; doi:10.3389/fpubh.2022.972766)
Supplement: Supplementary file 1 [file Table_1.DOCX]

**Table S1** Highly major MeSH headings/subheadings and their PubMed unique identifiers of source articles：Binary matrix that has source studies as columns and highly frequent terms as rows, 1 represents the study labeled by the MeSH, 0 represents no.

| No. | major MeSH headings/ subheadings | PubMed unique identifiers of source articles | | | | |
| --- | --- | --- | --- | --- | --- | --- |
|  |  | 24891052 | 12437452 | 25351668 | … | 31052295 |
| 1 | Skin Aging/ drug effects | 0 | 0 | 1 | … | 0 |
| 2 | Ultraviolet Rays/ adverse effects | 1 | 1 | 0 | … | 0 |
| 3 | Skin Aging/ radiation effects | 0 | 0 | 0 | … | 0 |
| 4 | Skin/ radiation effects | 0 | 1 | 0 | … | 0 |
| … | … | … | … | … | … | … |
| 30 | Low-Level Light Therapy/ methods | 0 | 0 | 0 | … | 0 |
| 31 | Skin Diseases/ drug therapy | 0 | 0 | 0 | … | 0 |
| 32 | Phototherapy/ methods | 0 | 0 | 0 | … | 0 |
